# Identification of a common risk haplotype for canine idiopathic epilepsy in the *ADAM23* gene

**DOI:** 10.1186/s12864-015-1651-9

**Published:** 2015-06-18

**Authors:** Lotta L. E. Koskinen, Eija H. Seppälä, Janelle M. Belanger, Meharji Arumilli, Osmo Hakosalo, Päivi Jokinen, Elisa M. Nevalainen, Ranno Viitmaa, Tarja S. Jokinen, Anita M. Oberbauer, Hannes Lohi

**Affiliations:** Research Programs Unit, Molecular Neurology, University of Helsinki, Helsinki, Finland; Department of Veterinary Biosciences and Department of Medical Genetics, University of Helsinki, Helsinki, Finland; Folkhälsan Institute of Genetics, Helsinki, Finland; Department of Animal Science, University of California Davis, Davis, California USA; Department of Clinical Veterinary Sciences, University of Helsinki, Helsinki, Finland

**Keywords:** Epilepsy, Dog, *ADAM23*, GWAS, Resequencing, SNP

## Abstract

**Background:**

Idiopathic epilepsy is a common neurological disease in human and domestic dogs but relatively few risk genes have been identified to date. The seizure characteristics, including focal and generalised seizures, are similar between the two species, with gene discovery facilitated by the reduced genetic heterogeneity of purebred dogs. We have recently identified a risk locus for idiopathic epilepsy in the Belgian Shepherd breed on a 4.4 megabase region on CFA37.

**Results:**

We have expanded a previous study replicating the association with a combined analysis of 157 cases and 179 controls in three additional breeds: Schipperke, Finnish Spitz and Beagle (p_c_ = 2.9e–07, p_GWAS_ = 1.74E-02). A targeted resequencing of the 4.4 megabase region in twelve Belgian Shepherd cases and twelve controls with opposite haplotypes identified 37 case-specific variants within the *ADAM23* gene. Twenty-seven variants were validated in 285 cases and 355 controls from four breeds, resulting in a strong replication of the *ADAM23* locus (p_raw_ = 2.76e–15) and the identification of a common 28 kb-risk haplotype in all four breeds. Risk haplotype was present in frequencies of 0.49–0.7 in the breeds, suggesting that *ADAM23* is a low penetrance risk gene for canine epilepsy.

**Conclusions:**

These results implicate *ADAM23* in common canine idiopathic epilepsy, although the causative variant remains yet to be identified. ADAM23 plays a role in synaptic transmission and interacts with known epilepsy genes, *LGI1* and *LGI2*, and should be considered as a candidate gene for human epilepsies.

**Electronic supplementary material:**

The online version of this article (doi:10.1186/s12864-015-1651-9) contains supplementary material, which is available to authorized users.

## Background

Epilepsy is a common neurological disease in both human and dog. Epilepsy is defined as recurrent, self-limited and unprovoked seizures, and can be classified according to the age of onset, the localization of seizure origin (focal or generalised), and aetiology. Epileptic seizures can be caused by trauma or structural abnormalities of the brain, or by metabolic disorders. The cause may also be directly genetic or idiopathic (IE) [1]. It affects approximately one to five percent of the human and canine populations, although it can be five to 10 times more common in some dog breeds [2].

Many human forms of epilepsy are considered to have a genetic basis though little is known about the underlying risk genes and variants for common forms of epilepsy despite increasingly powerful methodologies [3]. Rare Mendelian forms of the human disease have been associated with many ion channel subunits, although other types of gene pathways, related to neurodevelopment, mitochondria, and other cellular processes, are now being identified [3].

The high prevalence of epilepsy in many dog breeds suggests a strong genetic contribution [4] and could provide complementary resources to identify risk genes. Human and canine epilepsies share features, suggesting shared biological aetiologies. Indeed, several orthologues have been found in canine symptomatic recessively-inherited epilepsies [5–12]. However, only two genes have been associated with IE to date: *LGI2* in benign juvenile epilepsy in Lagotto Romagnolos [13], and the *ADAM23* risk locus in the Belgian Shepherd IE [14]. Although a genetic predisposition has been postulated in many breeds [4], the identification of risk genes has been challenging. Reasons include clinical and genetic heterogeneity and may relate to incomplete phenotyping, underpowered study cohorts, or incomplete resolution of the DNA marker panels.

In addition to the *LGI2* mutation [13], the rare breakthrough in canine IE comes from genetic studies in the Belgian Shepherd breed. The first microsatellite-based approach suggested six loci on CFAs 2, 6, 12 and 37 [15]. Genome-wide significance was later confirmed only at CFA37 by a low density genome-wide association study (GWAS) and finemapping with roughly 100 cases and 100 controls [14]. A seven-fold epilepsy risk was found with the homozygous risk haplotype in the *ADAM23* locus [14]. To confirm these earlier findings and to identify possible novel epilepsy loci, we re-genotyped a larger cohort of Belgian Shepherds with the latest high density single-nucleotide polymorphism (SNP) arrays and included three other breeds with IE: Finnish Spitz, Schipperke and Beagle. We report a shared risk in the four breeds and identify a common risk haplotype in the *ADAM23* gene by targeted resequencing and validation. This study emphasises the role of *ADAM23* in canine epilepsy, and proposes a role for the LGI-ADAM pathway in epilepsy across species.

## Results

### Epilepsy phenotypes

The seizure characteristics of idiopathic epilepsy in the studied breeds were described based on the detailed epilepsy questionnaire collected from the epileptic dogs in each breed. The seizure characteristics of Belgian Shepherds and Finnish Spitz have been described recently [14, 16] and are summarised for Schipperkes and Beagles in Additional file 1: Results. The defining characteristics of idiopathic epilepsy in the four breeds are presented in Additional file 2: Table S1. Briefly, IE in the four breeds show typical onset at early adulthood at three years of age (range 3 months – 9 years) and manifest both focal and generalised seizures. A high variability in seizure frequency is present in all breeds ranging from one reported episode in two years to several episodes per month. No evidence for sex predisposition was found in Belgian Shepherds or Schipperkes, but more affected males are reported in Beagles [17] and Finnish Spitz [16].

In addition to the questionnaire reports on seizure history, clinical examinations that included complete neurological exam, blood chemistry, cerebrospinal fluid (CSF) analyses, magnetic resonance imaging (MRI) and electroencephalography (EEG) were also performed in a selected group of dogs from three of the breeds to exclude possible external causes of seizures. None of the exams revealed abnormalities, whereas the EEG detected epileptic activity in some of the dogs supporting the diagnosis of IE. The clinical summaries of the Finnish Spitz and Belgian Shepherd examinations have been reported previously [14, 16]. The clinical results of 11 epileptic and five healthy control Schipperkes are presented in Additional file 1: Results. Clinical studies were not performed for Beagles.

### Breed-specific association studies

A total of 591 dogs in the four breeds were genotyped with the high-density SNP arrays. They formed distinct genetic populations according to the multidimensional scaling (MDS) plot drawn from the two top principal components of genetic variation (Additional file 3: Figure S1A). In Belgian Shepherds with two breed variants Groenendael (n = 118) and Tervueren (n = 137), 93 cases and 162 controls passed the diagnostic and genotype quality control criteria. This cohort is largely new having only 16 % overlap with our previous study that used the lower density arrays [14]. The breed variants are closely related but represent separate clusters in the MDS plot (Additional file 3: Figure S1B). The genomic inflation factor λ was 0.81. GWAS analysis revealed only one genome-widely significant locus, confirming the previously identified association on CFA37 at 14.5–17.5 Mb with the best associated SNP BICF2P1131874 at 15111724 bp (p_raw_ = 3.57E–08, p_GWAS_ = 3.78E–03) (Additional file 4: Figure S2A).

The Schipperke cohort with 67 IE cases and 70 controls formed a single population in the MDS plot analysis (Additional file 3: Figure S1C) as expected since the majority of the dogs came from one large pedigree (Additional file 5: Figure S3). Despite correcting for the relatedness using mixed model approach, no significant association was revealed in the GWAS (Additional file 4: Figure S2B). The genomic inflation factor λ was 0.85.

The Finnish Spitz cohort including 61 cases and 68 controls, clustered together in the MDS plot (Additional file 3: Figure S1D). GWAS did not reveal any significant association (Additional file 4: Figure S2C). The genomic inflation factor λ was 0.81.

The Beagle cohort consisted of 29 IE cases and 41 controls and represented two clearly separate genetic populations in the MDS plot (Additional file 3: Figure S1E). The Beagle samples originated from Finland (60 %), Germany (30 %) and Sweden (10 %). Most of the Finnish dogs clustered separately from the German dogs, although some overlap was present. Three Finnish and four Swedish Beagles clustered together with the German Beagles, and three Swedish Beagles clustered together with the Finnish Beagles. The majority of the Finnish Beagles belonged to the Finnish hunting line, which has been maintained separately from other Beagles to maintain the hunting skills. The two genetic subpopulations seen in the MDS plot represent this population substructure (Additional file 3: Figure S1E). GWAS revealed no significant association in Beagles (Additional file 4: Figure S2D), even in an independent analysis of the German and Finnish hunting lines (data not shown). The genomic inflation factor λ was 0.81.

### Across breed GWAS

**Fig. 1:**
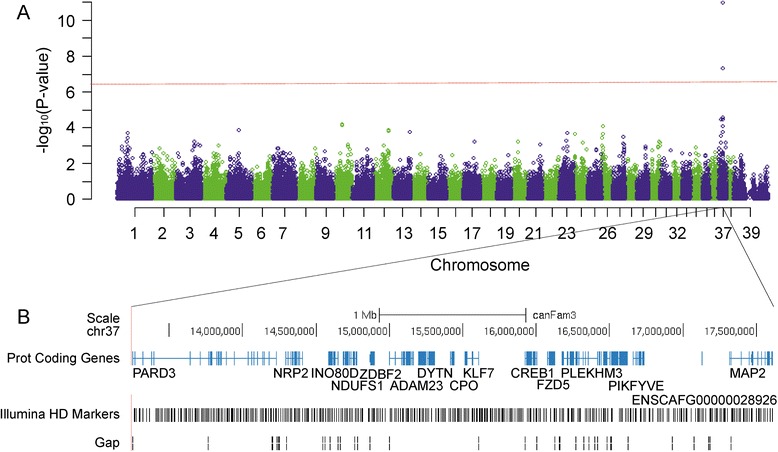
Genome-wide association study across four breeds and gene content of the associated locus on CFA37. **a**. Genome-wide association study including Belgian Shepherds, Schipperkes, Finnish Spitz, and Beagles highlights a single locus on CFA37. The SNPs showing the strongest association with IE are located in the *ADAM23* gene. The red line indicates genome-wide significance level of p-values. **b**. The UCSC-based gene content of the 4.4 Mb critical region on CFA37, which was targeted and resequenced. The figure also includes the genotyped HD markers in the locus and annotation gaps in the CanFam3.1 reference

The GWAS data from the four breeds were analysed jointly for possible common risk variants. An intronic SNP BICF2P1131874 in the *ADAM23* gene on CFA37 at 15111724 bp showed the strongest association signal (p_raw_ = 1.04e–11, p_GWAS_ = 6.17e–07, λ 0.87) (Fig. 1a, Additional file 4: Figure S2E). When the combined data were analysed without Belgian Shepherds, another intronic SNP in the *ADAM23* gene showed strong association (BICF2P1290526 at 15093174 bp, p_c_ = 2.93e–07, p_GWAS_ = 1.74e–02, λ 1.11) (Additional file 4: Figure S2F). These results suggest a shared risk locus exists across the four breeds.

### Targeted resequencing of the *ADAM23* locus in Belgian Shepherds

Our two GWAS experiments in Belgian Shepherds (the present and the previous study) support the strongest association within the *ADAM23* gene. Therefore, we selected the two highest associated risk variants within *ADAM23* to define the risk haplotype for 12 IE cases and 12 controls with opposite homozygous haplotypes. A 4.4 Mb region spanning *ADAM23* was captured and resequenced. Resequencing data of CFA37 was of high quality with an average 100x sequence coverage in each sample in the target region (Additional file 6: Table S2, Fig. 1b). In total, 5934–9463 single-nucleotide variants (SNVs) and 2922–4206 insertion-deletion variants (indels) were identified in each dog (Additional file 6: Table S2).

However, only 37 variants showed opposite genotypes in the risk and non-risk haplotypes, including 29 SNVs and eight indels unique to all 12 cases. These 37 variants cover a 96 kb interval within *ADAM23* (15085438–1518443 bp). These variants included one coding variant (*c.1159G > A*, p. R387H at 15113940 bp) that was identified in our previous Sanger sequencing experiment and was used to define the risk haplotype [14]. All the other variants identified were intronic to *ADAM23* with 13 known variants from dbSNP.

### Validation reveals a shared six-variant risk haplotype across breeds

We prioritised 25 of the 37 variants discovered in resequencing for a validation in a larger cohort using conservation scores in the public databases (Ensembl and UCSC) (Additional file 7: Table S3). These 25 variants included one coding variant (*c.1159G > A*, p. R387H, 15113940 bp) and 24 intronic variants in *ADAM23*. In addition, we added a second coding variant (*c. 1158C > T*, p. R387C, 15113939 bp), which is rare in Belgian Shepherds, but was detected in sequencing samples from other breeds in our previous study (unpublished result) [14]. *ADAM23* intronic variant BICF2P1290526 (at 15093174 bp) was included in the validation, as it showed the strongest association signal in the combined GWAS without Belgian Shepherds. In total, 27 variants were genotyped in 640 dogs across four breeds (Belgian Shepherds, Schipperkes, Finnish Spitz and Beagles). One variant appeared monomorphic (15181443 bp) and was excluded from further analyses.

The genotyping data was analysed for association in individual breeds and all breeds combined. We found that the coding variant (*c.1159G > A*, p. R387H) was associated with IE in Belgian Shepherds (p_raw_ = 2.59e–11) and Finnish hunting Beagles (p_raw_ = 0.008), but not in German Beagles (p_raw_ = 0.95), Schipperkes (p_raw_ = 0.98) or Finnish Spitz (p_raw_ = 0.98) (Additional file 7: Table S3). The second coding variant (*c.1158C > T*, p. R387C) was associated with IE in Schipperkes (p_raw_ = 0.005) and Finnish Spitzs (p_raw_ = 0.05), but not in Finnish (p_raw_ = 0.98) or German Beagles (p_raw_ = 0.60), or Belgian Shepherds (p_raw_ = 0.2). The minor allele (T) frequency was only 0.006 in Belgian Shepherds (Additional file 7: Table S3).

**Fig. 2:**
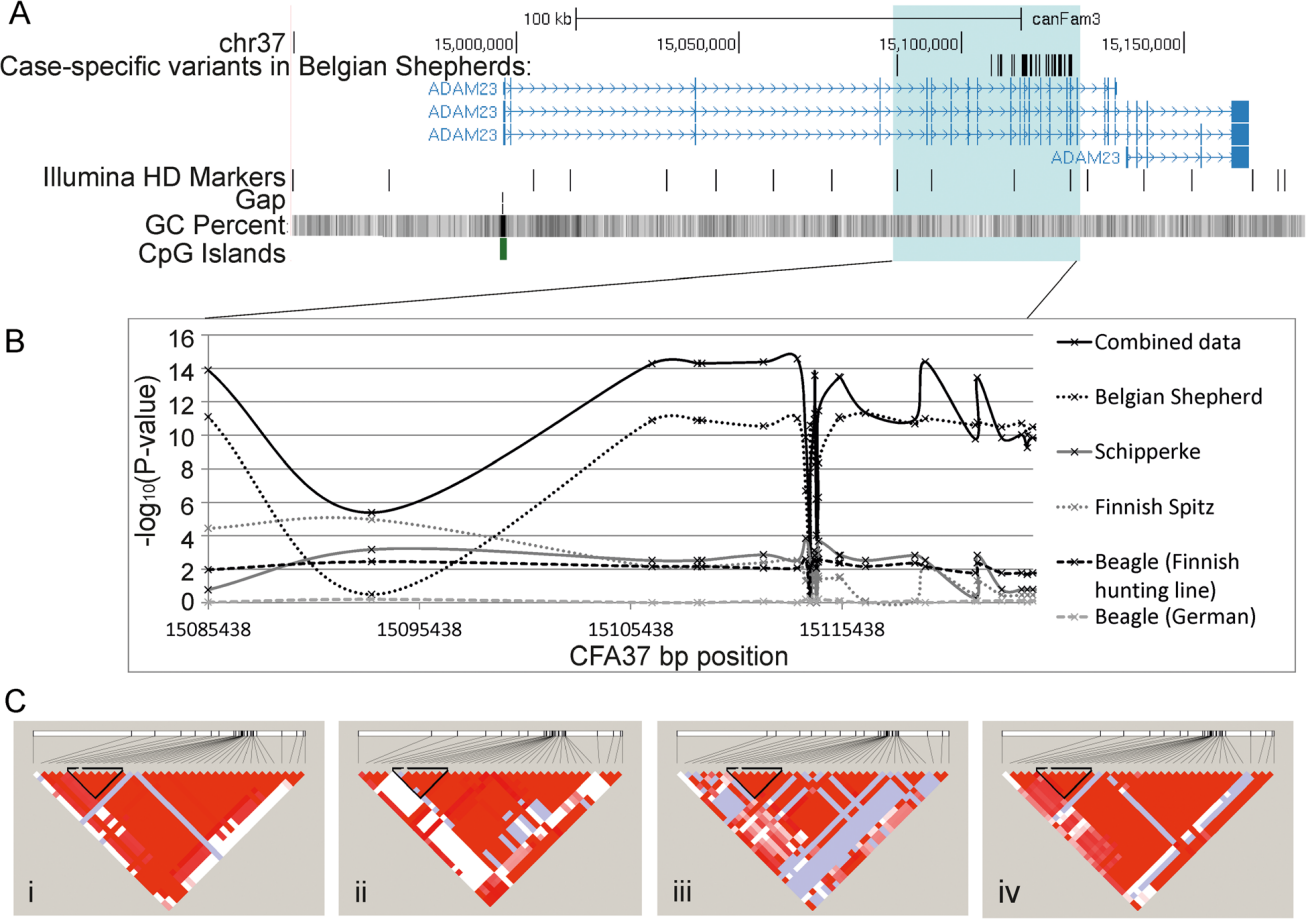
The variants identified in the resequencing and associated with idiopathic epilepsy are located in *ADAM23*. **a**. The 37 case-specific variants identified in Belgian Shepherds by targeted resequencing were located within a 39-kb region of *ADAM23* and span exons 5–17 highlighted with blue. The entire *ADAM23* region spanning 14,970,211–15,175,391 bp on CFA37 based on the UCSC Genome Browser on dog (CanFam3.1). Thirteen Illumina HD markers are within the *ADAM23* gene, and four within the 39-kb region. There is a gap in the reference sequence in the 5’ end *ADAM23*, and the exon 1 sequence has high GC content. **b**. Association results of the 26 variants at the target region on CFA37 in Belgian Shepherds, Schipperkes, Finnish Spitzs, Beagles (Finnish hunting and German sub-populations), and combined data. The coding variants at 15113939 and 15113940 bp show inconsistent association between the breeds. **c**. There is strong linkage disequilibrium in the *ADAM23* gene region in the four associated breeds: (i) Belgian Shepherds, (ii) Schipperkes, (iii) Finnish Spitzs and (iv) hunting Beagles from Finland. The most consistent association across breeds was identified for a six-variant risk haplotype spanning a 28-kb region containing exons 5–11 outlined with black

A majority of the intronic SNPs showed significant associations in individual breeds (Additional file 7: Table S3). In the combined analysis of all four breeds, intronic variants both upstream and downstream of exon 12 showed strong association with the lowest p-value at 15113325 bp (p_cmh_ = 2.76e–15, OR 2.93, 95 % CI 2.23–3.85) (Fig. 2b; Additional file 7: Table S3).

Haplotype analysis using Haploview indicated a strong linkage disequilibrium between the 26 variants (Fig. 2c) defining a risk haplotype that spanned almost the whole genotyped region (~96 kb) in each breed. The risk haplotypes were similar but not identical across breeds. Breed-wise comparisons revealed a shared 28-kb six-variant risk haplotype (*T-C-del-del-G-G)* encompassing the exons 5–11 of *ADAM23* between 15085438 and 15113325 bp (Table 1). Dogs homozygous for the risk haplotype had the highest risk of epilepsy with OR varying from 3.33 to 12 across associated (*p* < 0.05) breeds (Table 1). For the four breeds having a significant risk haplotype, fewer than 10 % of cases did not carry that risk haplotype.

**Table 1:** Haplotype analysis revealed a common risk haplotype for idiopathic epilepsy. A risk haplotype shared between the breeds was identified between 15085438 and 15113325 bp on CFA37. It consisted of alleles *T-C-del-del-G-G* at 15085438, 15106446, 15108593, 15108802, 15111724 and 15113325 bp in *ADAM23*

Breed	Risk haplo freq. cases	Risk haplo freq. controls	p-value	OR (95 % C.I.)	P-value (homozygosity)	OR (95 % C.I.) (homozygosity)	Cases/controls without risk haplotype
Belgian Shepherd	0.75	0.49	4.3e–11	3.2 (2.3–4.6)	4.1e–10	4.6 (2.8–7.6)	10 %/28 %
Schipperke	0.81	0.65	0.003	2.3 (1.3–4.0)	0.0006	3.3 (1.7–6.7)	6 %/9 %
Finnish Spitz	0.89	0.67	1.6e–05	4.1 (2.1–8.2)	4.9e–05	4.9 (2.2–10.8)	2 %/12 %
Beagle (Finnish hunting)	0.96	0.7	0.006	11.5 (1.4–93.1)	0.009	12 (1.4–106.2)	0 %/12 %
Beagle (German)	0.63	0.65	0.9	0.9 (0.3–2.6)	0.9	1.1 (0.3–4.6)	19 %/13 %

*haplo freq* haplotype frequency, *OR* odds ratio, *C.I.* confidence interval

### Uncovering the missing upstream sequence for *ADAM23*

In the absence of a compelling causative variant in the coding region of *ADAM23*, we hypothesised that the variant was in the regulatory region of the gene. However, the dog reference sequence (CanFam3.1) has a gap of unknown size in the regulatory region upstream of exon 1 of the *ADAM23* gene (Additional file 8: Figure S4). Additionally, the sequence in the 5’ end of the *ADAM23* gene, including exon 1, is highly GC-rich (>80 %), challenging conventional mutation screening. For example, there is a 6-bp sequence (CCCCTG) repeated seven times immediately upstream of exon 1 (Additional file 8: Figure S4). To enable efficient study of this 5’ regulatory region, we aimed to fill the gap using a BAC clone from the target region and PacBio sequencing technology. We found a 190-bp GC-rich (87 %) sequence with simple repetitive elements that has expanded the canine reference sequence for this gene (Additional file 8: Figure S4). This 5’ region upstream of exon 1 could not be included in the capture design and while exon 1 was included in the design, it did not work in the capture experiment, possibly due to the very high GC-content of the region (Fig. 2a).

## Discussion

This study significantly expands from our previous IE studies in Belgian Shepherds [14, 15] to achieve four major goals. First, we sought to confirm the association at CFA37 and possibly reveal other loci by replicating our original low resolution study using high resolution GWAS in a larger independent cohort. Second, we assessed three other IE breeds (Schipperke, Finnish Spitz, and Beagle) with reasonable sample size by GWAS to map new IE loci. Third, we undertook a combined analysis of the data for the four breeds to identify shared IE loci. Finally, we aimed to refine the associated region and identify causative variants by targeted resequencing.

Our findings provide strong genetic evidence for the role of *ADAM23* in canine IE. The Belgian Shepherd data with increased resolution and sample size confirmed the association to *ADAM23* on CFA37 but did not reveal new loci at a genome-wide significant level. A combined analysis of the three other breeds using high resolution GWAS also highlighted the *ADAM23* gene though breed-specific associations were not revealed. Our previous attempt to demonstrate the *ADAM23* locus involvement in those breeds [14] failed because the selected SNPs were uninformative to detect association in these breeds. Our targeted resequencing of the large 4.4 Mb locus uncovered a set of new case-specific variants found only in the *ADAM23* gene, and a replication study with those variants revealed a strongly associated shared risk haplotype in all four breeds. The 28-kb risk haplotype consisting of intronic variants lies in the middle of the *ADAM23* gene. These results implicate *ADAM23* in IE and suggest that the causative variant (s) yet to be found resides within the risk haplotype or adjacent to the gene outside the coding regions. The two missense variants affecting the R387 amino acid in exon 12 of the *ADAM23* gene show conflicting association in the studied breeds suggesting this is not the causal mutation. The sequence gap at the 5’ region of *ADAM23* and poor resequencing coverage in the GC-rich exon 1 did not permit definitive assessment of the 5’ regulatory region of the gene. We did however, uncover the 190 bp sequence gap immediately upstream of *ADAM23* in a BAC clone. This will help the future characterization of the role of the 5’ regulatory region in canine IE.

While most affected dogs carried the risk haplotype, it was also commonly found in control dogs with frequencies of 0.49 to 0.70 depending on the breed. These results suggest a low penetrance of the seizure predisposing variant in *ADAM23*. The high frequency of the risk haplotype combined with low penetrance may partly explain why IE is so common in many dog breeds despite active prevention efforts in the breeding programs. Accurate estimation of penetrance may be adversely affected by imperfect diagnosis of mild cases that are now in the control group. Identification of the causative variant (s) will permit more precise estimations in future experiments.

*ADAM23* is an excellent candidate gene for IE. It interacts with known IE genes, *LGI1* and *LGI2* [13, 18, 19]. A truncating mutation in the *LGI2* gene prevents its secretion in neurons and causes recessively inherited benign juvenile epilepsy in the Lagotto Romagnolo breed [13]. *LGI2* is a paralog of *LGI1* which is the causal gene for ADTLE/ADPEAF (autosomal-dominant temporal lobe epilepsy/autosomal dominant partial epilepsy with auditory features, MIM# 600512), a non-remitting epilepsy in humans with onset typically in adolescence or early adulthood [20]. Both LGI1 and LGI2 are neuronally secreted proteins [21], and interact with ADAM neuronal membrane proteins, such as ADAM11, ADAM22 (post-synaptic) and ADAM23 (pre-synaptic) [18]. ADAM23 is expressed in the central nervous system, and high expression levels have been detected in the cerebral cortex pyramidal cells and in the CA1 and CA3 pyramidal cells of the hippocampus, as well as in the cerebellar Purkinje cells [18, 22, 23]. ADAM23 is the primary receptor for LGI1, and needed for LGI1-mediated dendritic branching [23]. The function of the ADAM23-LGI2 complexes is currently being studied.

A complete knockout of *ADAM23* in mice results in ataxia, tonic-clonic seizures, failure to thrive and death within two weeks from birth [23, 24]. Heterozygous mice appear otherwise normal but have a lower seizure threshold [23]. A reduction in the ADAM23-mediated LGI1-stimulation of neurite outgrowth in the central nervous system was suggested to contribute to epilepsy, although other yet unknown ADAM-LGI-mediated alterations may exist [23].

## Conclusions

Our genetic data implicates *ADAM23* as a common risk gene for idiopathic epilepsy in four unrelated dog breeds and further replication studies in additional breeds are ongoing. Our data suggest causal variation with low penetrance in the regulatory region of the gene. The identification of the causal variation (s) and further functional studies are warranted to improve the understanding of the ADAM23-mediated molecular mechanisms in the brain and their role in seizure susceptibility.

## Methods

### Sample collection and phenotyping

Whole blood samples were collected from privately owned dogs with the owner’s consent mainly from Finland but also from USA, Germany, Sweden, Poland, Switzerland and Australia. Buccal swab samples were collected from 108 Belgian Shepherds from USA and four Schipperkes from UK. The samples were recruited through breed clubs, scientific collaborators, veterinarians, and advertisement in various dog fancier forums. The samples were stored at the Dog DNA bank at the University of Helsinki along with the dogs’ health and pedigree information. Collection of samples was approved by the Animal Ethics Committee at the State Provincial Office of Southern Finland (ESLH-2009-07827/Ym-23 and ESAVI/6054/04.10.03/2012) or by the University of California, Davis Institutional Animal Care and Use Committee.

The diagnosis of idiopathic epilepsy was based on a 10-page epilepsy questionnaire available in multiple languages [25]. The questionnaire was required from all dogs with owner-reported seizures unless sufficient information was available through phone or e-mail interviews with the dog owner, or dog health information updates to the research group by the dog owner. The diagnosis was established by the following criteria: at least two seizure episodes at least two months apart, no other known conditions or events which could predispose to seizures (e.g., untreated metabolic diseases, brain tumours, head trauma, or intoxication), age of onset between six months and five years. Dogs with lower or higher age of onset were included if findings in general clinical and neurological examination, complete blood count, blood biochemistry and MRI supported the diagnosis of IE.

The control dogs were older than seven years of age and had never had epileptic seizures or other owner reported chronic diseases. The health status of the control dogs was confirmed by contacting the owner by phone, e-mail or mail. The controls were matched to the cases according to breed, breed variant, and country of origin.

A large proportion of the Schipperke cohort consisted of a pedigree of closely related individuals, including concordant and discordant sibling pairs, their parents, grandparents, half siblings, but also few unrelated cases and controls. The Schipperke pedigree is presented in Additional file 5: Figure S3.

### Clinical examination

Eighteen Schipperkes (11 cases, five controls and two with unknown status) were clinically studied including general clinical and neurological examination, complete blood count, blood biochemistry, 20-min interictal EEG, MRI, and CSF analysis. The clinical studies were performed at the Referral Animal Neurology Hospital Aisti, Vantaa, Finland as previously described [14, 16].

### DNA extraction

Genomic DNA was extracted from the whole blood samples and buccal swabs of four Schipperkes using the Puregene DNA Purification Kit (Gentra Systems) or Chemagic Magnetic Separation Module I (Chemagen Biopolymer-Technologie AG, Baeswieler, Germany) according to the manufacturer’s instructions. The Qiagen DNA Blood Mini Kit (QIAGEN Inc., Valencia, CA) was used for a portion of the Belgian Shepherd samples from the US [26]. The remaining Belgian Shepherd DNA samples from the US were extracted from buccal swabs as previously reported [27].

### Power analysis

To replicate the previous findings in Belgian Shepherds on CFA37, we estimated the required sample size using the Genetic Power Calculator [28]. The parameters used in the calculations were derived from our previous article [14]: high risk allele frequency and marker allele frequency 0.49, disease prevalence 0.10, genotypic relative risk Aa 3, genotypic relative risk AA 5, and D’ 0.8. To have an 80 % power with 0.05 significance level in case–control allelic 1 df test, 77 cases were needed. The statistical power of our study materials were as follows: Schipperkes 0.72, Finnish Spitz 0.67, Beagles (total) 0.46, Beagles (Finnish hunting subpopulation) 0.29, and Beagles (German subpopulation) 0.23. The statistical power in the whole replication material was 0.98.

### Genome-wide association study

A total of 591 dogs were included in the genome-wide analyses from 4 dog breeds: Belgian Shepherd (Groenendael and Tervueren), Schipperke, Finnish Spitz, and Beagle, and 640 dogs were included in the finemapping of the *ADAM23* locus. The Belgian Shepherd sample cohort used had a 16 % overlap with the samples used in our previously published study [14]. The GWAS and finemapping cohort were mainly overlapping with each other. The Belgian Shepherd cohort included in the finemapping effort partially overlapped with the cohort used in our previous study [14]. The number of samples from each breed is presented in Table 2.

**Table 2:** The number of samples included in the genome-wide association study (GWAS) and the validation of the variants on CFA37

Breed	GWAS		CFA37 target region finemapping	
	Cases	Controls	Cases	Controls
Belgian Shepherd	93	162	127	176
Groenendael	57	61	73	66
Tervueren	36	101	54	110
Schipperke	67	70	68	70
Finnish Spitz	61	68	61	68
Beagle	29	41	29	41
German subpopulation	15	16	15	16
Finnish hunting line	14	25	14	25
Total	250	341	285	355

The samples were genotyped with the Illumina Canine HD Bead chips (Illumina, Inc., San Diego, CA, USA) for more than 173,000 SNPs across all canine chromosomes. The Beagle, Schipperke, and Finnish Spitz samples were genotyped at the Centre National de Génotypage (Paris, France) as part of the EU-funded LUPA project [29], and the Belgian Shepherd samples were genotyped at GeneSeek (Lincoln, NE, USA). The genotype data was analysed with GenABEL v. 1.6–7 [30] in R v. 2.13.0. The chromosomal positions of the SNPs were based on the CanFam3.1 build. The data was filtered for minor allele frequency > 0.05, genotype call rate > 0.95, individual call rate > 0.95 and Hardy-Weinberg equilibrium *p* > 0.0001 in the controls. MDS plots were produced for each genotyped breed to identify outliers and population stratification. Identity-by-state values were calculated for each pair of individuals to control for duplicate samples and known relatedness. The GWAS were performed using mixed model approach in GenABEL [30, 31]. Mixed models test was selected because it corrects for the relatedness between the genotyped individuals. The estimation of polygenic model was performed using the polygenic option in GenABEL [32]. If the genomic inflation factor λ was >1, corrected association p-values were used. In addition, association p-values were adjusted using Bonferroni correction to control for multiple testing by multiplying the association p-values by the number of SNPs included in the analyses.

The quality control and GWAS were performed separately for each breed. For breeds with more than one breed variant or line, the GWAS analyses were performed using the variant or line as strata, and the analyses were also performed separately in each variant or line. Combined analysis of all four breeds was performed using breed as strata.

### Targeted resequencing in Belgian Shepherds

A 4.4 Mb locus on CFA37 was selected for a sequence capture and resequencing experiment (Fig. 1b). We selected 12 Belgian Shepherd cases and 12 healthy Belgian Shepherd controls homozygous with respect to the risk- and non-risk haplotypes based on our previous GWAS results [14]. The risk and non-risk haplotypes were defined based on the alleles for BICF2P890779/rs24025367 at 15124213 bp, and a p. R387H (*c. 1159G > A*) variant in *ADAM23* exon 12 at 15113940 bp. The mutation nomenclature is based on GenBank accession XM_844759.3 (*ADAM23*) with nucleotide one being the first nucleotide of the translation initiation codon ATG. The region between 13,250,000 and 17,612,000 bp on CFA37 was selected for the sequence capture, because it contains the associated locus. There is a gap in the 5’ end of the *ADAM23* gene, and this region was thus excluded from this original sequence capture design. The 5’ region was sequenced using a BAC clone (see PacBio method below). The SeqCap EZ Developer Library capture assays were designed by Roche-Nimblegen (Madison, WI, USA) for the CanFam3.1 build, and the in-solution sequence capture and paired-end sequencing were performed at the Institute for Molecular Medicine Finland (FIMM) Sequencing facility (Helsinki, Finland). The sequencing was performed with the HiSeq2000 instrument (Illumina, Inc., San Diego, CA, USA).

The data was analysed with a custom-developed bioinformatics pipeline [33]. On average, 8523875 paired-end reads were generated with read lengths of 93 bp for each sample. Quality check was performed on the raw data with FASTX-Toolkit [34] to remove the bases incorrectly called by the sequencing machine. For this, base pairs below a base call accuracy of 99 % i.e., bases with Phred scores < Q20 were trimmed to decrease false-positive variants during variant calling. The quality controlled paired-end reads were aligned to the canine reference genome (CanFam.3.1) using BWA v. 0.6.1 alignment tool with default parameters [35]. An average of 95.75 % of the reads were mapped to the reference genome for each sequenced sample. After mapping, the reads that mapped to the targeted region were extracted followed by the removal of potential PCR duplicate reads using SAMtools v. 0.1.18 [36]. This has retained an average of 50.75 % of the reads mapped in the targeted region of 4 Mb on CFA37.

Fold coverage for each base in the target region was calculated using unique reads. The average coverage for each sample in the target region was 100-fold. Less than 1.5 % of the targeted bases were not covered with at least one read whereas 83 % of the targeted bases were covered with at least 50X coverage, which signifies the high quality of the data. A region between 14996848 and 14997400 bp, spanning exon 1 of the *ADAM23* gene was not covered in any of the samples. Coverage statistics for all the sequenced samples were calculated using GATK v. 2.1-12-ga99c19d [37, 38].

Local Realignment around potential insertion-deletion sites and base quality scores recalibration were implemented using GATK [37, 38] and fix mate-pair information using SAMtools [36] to improve the quality of the sequence data before variant calling. We aimed for maximum sensitivity to detect variants in the targeted region and therefore considered the union of variant calls from two variant calling algorithms; SAMtools [36] and GATK [37, 38]. In total, we identified an average of 7955 SNPs and 3650 indels in each sample (Additional file 6: Table S2). The identified variants were compared to the NCBI SNP database (dbSNP build 131). On average, 18 % of the variants were known, and 82 % were novel in each sample. Furthermore, variants were annotated to the Ensembl gene set to find the location in the reference genome using ANNOVAR [39].

Thirty-seven variants (29 SNVs, eight short indels) with opposite genotypes in the individuals with the risk-and non-risk haplotypes were identified from the data for further validation (Additional file 9: Table S4). The variants were confirmed by visual inspection of the reads and variant calls with the Integrative Genomics Viewer [40, 41].

### PacBio sequencing

A BAC clone CHORI82_R4_266G10, which spans the genomic region of *ADAM23* on CFA37 (at 14898298–15055982 bp) was sequenced using PacBio sequencing technology (Pacific Biosciences, Menlo Park, CA, USA) at DNA Sequencing and Genomics laboratory, Institute of Biotechnology, University of Helsinki, Finland. The BAC clone was provided by The BACPAC Resource Center (BPRC) (Children’s Hospital Oakland Research Institute, Oakland, California, USA) in DH10B *E. Coli* host. The clone was amplified and cultured according to the provider. BAC clone DNA was extracted with the QIAGEN Large-Construct Kit (Qiagen, Hilden, Germany). The clone (25 μg DNA) was sequenced using the PacBio RS sequencing platform using P5 polymerase and C3 chemistry (P5-C3). Total of 529.8 Mbp PacBio reads were assembled using HGAP.3 (Hierarhical Genome Assembly Process) implemented in SMRT Analysis. The complete circular BAC clone, in length of 212 184 bp, was sequenced at 2200x coverage.

### Validation and haplotype analysis

Twenty-five case-specific variants (20 SNVs and five indels) identified in the resequencing were selected for validation in larger sample sets (Table 2, Additional file 9: Table S4). Variants with high conservations scores were prioritized for validation. The conservation was evaluated through GERP scores [42] obtained from Ensembl [43], and PhastCons and Phylop scores available through UCSC Genome Browser [44, 45]. Two additional SNVs were included in the genotyping: BICF2P1290526 at 15093174 bp which showed the strongest association signal in the *ADAM23* gene in the combined GWAS without Belgian Shepherds, and the p. R387C (*c. 1158C > T*) variant at 15113939 bp in exon 12 of *ADAM23* which we had identified in our previous Sanger sequencing experiments (unpublished result) [14]. Thus, a total of 27 variants were included in the validation.

A Sequenom iPlex Gold (Sequenom, Inc, San Diego, CA, USA) experiment was designed for 21 variants (Additional file 9: Table S4). Six additional variants were genotyped by Sanger sequencing because they did not fit in the iPlex pool. A total of 640 samples from four dog breeds were included in the validation experiment based on the association results on CFA37 in the GWAS (Table 2). The Sequenom experiment was designed and the genotyping with the MALDI-TOF mass spectrometer were performed at FIMM Technology Centre (Helsinki, Finland), and yielded a 100 % call rate. The accuracy of the genotypes was controlled by using duplicate samples on each plate, and checking for Mendelian inconsistencies when possible. The Sanger sequencing was performed using standard protocols. Sequencing was carried out at the FIMM Technology Centre (Helsinki, Finland) and the genotypes were determined using Sequencher 5.1 sequence analysis software (Gene Codes Corporation, Ann Arbor, MI, USA).

The genotypes were analysed for association using PLINK v. 1.07 options for chisq test for allelic and genotypic associations [46, 47]. The haplotype blocks were determined with Haploview [48]. The analyses were done for each breed and breed variant or line separately. Combined association analysis was performed using the Cochran-Mantel-Haenzel-test to control for the differences in the odds ratios between breed clusters. The six-variant haplotypes for each individual were estimated using PLINK v. 1.07 [46, 47].

#### Availability of supporting data

The GWA data sets supporting the results of this article are available in the Labarchives repository, https://mynotebook.labarchives.com/share/Koskinen_et_al_BMC_Genomics/MS4zfDkxMDk0LzEvVHJlZU5vZGUvMTUzMDM2NTk5NHwzLjM=. The *ADAM23* 5’ gap sequence data is available in the European Nucleotide Archive, LN849740, http://www.ebi.ac.uk/ena/data/view/LN849740.

## Additional files

Additional file 1: Results. Epilepsy questionnaires and clinical results. Additional file 2: Table S1. The main characteristics of idiopathic epilepsy in four dog breeds based on owner-filled epilepsy questionnaires collected in 2003–2013. Additional file 3: Figure S1. Multidimensional scaling plots. Additional file 4: Figure S2. Results of the genome-wide association study (GWAS). Additional file 5: Figure S3. The Schipperke pedigree. Additional file 6: Table S2. A summary of the results of the resequencing of a 4.4 Mb locus on CFA37 in 12 Belgian Shepherd cases and 12 controls with epilepsy. Additional file 7: Table S3. Association results of the validation of the 26 *ADAM23* variants in 285 cases and 355 controls with idiopathic epilepsy. Additional file 8: Figure S4. Overview of the *ADAM23* 5’- region and DNA sequence. Additional file 9: Table S4. Variants belonging to the epilepsy risk haplotype in Belgian Shepherds identified in the targeted next-generation sequencing.
