# Supplementary material for: Identification of a common risk haplotype for canine idiopathic epilepsy in the ADAM23 gene
Source: BMC Genomics. 2015 Jun 18;16(1):465. doi: 10.1186/s12864-015-1651-9 (PMC4470040; doi:10.1186/s12864-015-1651-9)
Supplement: Additional file 1: Results. — Epilepsy questionnaires and clinical results. [file 12864_2015_1651_MOESM1_ESM.pdf]

Identification of a common risk haplotype for canine idiopathic epilepsy in the *ADAM23* gene

Koskinen *et al.*

Additional information

Additional results

Epilepsy questionnaires

The seizure characteristics of idiopathic epilepsy (IE) in the studied breeds were described based on detailed epilepsy questionnaires collected from epileptic dogs in each breed. The seizure characteristics of Finnish Spitzs and Belgian Shepherds have been presented recently [1, 2]. A summary of the questionnaires is presented in Additional Table 1. The median age of epilepsy onset ranged between 1.3 and three years, and the dogs had a median of 2-12 seizures per year, depending on the breed, Finnish Spitzs having the smallest number of seizures, and Belgian Shepherd Tervuerens having the highest number of seizures. The median seizure duration was 3-10 minutes, but there was a large range within each breed. In each breed, many owners reported pre-ictal symptoms in their dogs, for example seeking out the owner, restlessness and nausea. Stiffening of limbs and neck, muscle fasciculation and tremor were the most typical ictal signs in all the breeds. A majority of the dogs had impaired consciousness during the ictus in all breeds. Fatigue was the most typical post-ictal symptom, and a majority recovered in less than 30 minutes. Medical treatment was considered effective, i.e., seizure frequency was reduced at least by half compared to pre-medical seizures, in Finnish hunting line Beagles, Finnish Spitz and Belgian Shepherds. In Schipperkes, seizures had reduced by at least half in 46% of the cases. No information was available for the Beagles belonging to the German subpopulation. The follow-up time of the medication efficacy was variable, and likely not sufficient to draw conclusion about medication efficacy.

Schipperkes

Epilepsy questionnaires were collected from 62 schipperkes for this study, and 56 fulfilled the inclusion criteria. The main results are presented in Additional Table 1. The questionnaires were not completely filled out by all dog owners, and the results reported here are calculated based on the available data. The median age of onset was 1.3 years, and the dogs had a median of four seizures per year. Fourteen (25%)

dogs had had more than one seizure within 24 hours. The median seizure duration was four minutes (range 0.5-120 min). Stress and changes in hormone levels were the most typical seizure-predisposing factors. Pre-ictal symptoms were recognised in 34% of the dogs based on the dog's behavior (seek out the owner, restlessness) and autonomic symptoms (nausea). The most typical symptoms during the ictus were manifested as tonic-clonic, tonic, or clonic movements, and/or tremor. Behavioural symptoms were recognised in dogs as seeking out the owner and fear. The most common autonomic symptoms were salivation, urination, staring, and dilation of pupils. Continuous changes of posture, or chewing movements were also typical. A majority of the dogs recovered from the seizure in less than 15 minutes, the most common post-ictal symptoms being fatigue and confusion.

Thirty-two percent of the dogs were on anti-epileptic medication. Twelve dogs were treated with phenobarbital monotherapy, four with phenobarbital and potassium bromide, and one with phenobarbital and clonazepam. The medication had completely stopped the seizures in 15% (2/13) of dogs and reduced the number of seizures in 62% (8/13) of dogs. Thirty-one percent (4/13) of the dogs also had less severe seizures than prior to the introduction of the anti-epileptic therapy. The medication had not reduced the number of seizures of three (23%) dogs. Eight (62%) dogs had side effects, the main symptoms being increased thirst and hunger, fatigue and sleepiness. Four dogs (31%) had no side effects. Two dogs had received valerian as an alternative treatment, but the effectiveness of the treatment was uncertain.

Beagles

Epilepsy questionnaires were collected from 35 Beagles (Additional Table 1). For comparison, the results are presented separately for the Finnish hunting Beagles and German Beagles. The two subgroups were interviewed using different questionnaires, and the data of the group consisting of mainly German dogs is not fully comparable with the other questionnaire data. Sixteen Finnish hunting Beagles fulfilled our inclusion criteria. The median age of epilepsy onset was 2.9 years, and the dogs had a median of ten seizures per year. Cluster seizures were not common; only one dog had experienced more than one seizure within 24 hours. The median seizure duration was six minutes, but the duration ranged between 0.5 and 20 minutes. Excitement and stress were the most common factors which increased the risk of seizure. For three dogs, no seizure-triggering factors were recognised. Pre-ictal symptoms were recognised in six (40%) dogs mainly based on behavior (seeking out the owner, restlessness) and autonomic symptoms (nausea). These symptoms were seen less than 30 minutes before the ictus started. The most typical ictal symptoms were motor, manifesting as tonic-clonic, tonic, clonic movements and tremor of limbs and the whole body. Behavioural symptoms were recognised as seeking out the owner and fear. The dogs also had autonomous

symptoms typically manifesting as staring, salivation, and dilatation of pupils. Automatisms such as changing posture and chewing movements were also reported. A majority of the dogs recovered from the seizure in less than twenty minutes, but for some dogs the recovery took several hours. Fatigue and thirst were the most typical post-ictal symptoms.

Fifty-six percent (9/16) of the dogs were on anti-epileptic medication and all were treated with phenobarbital monotherapy. Information about the effectiveness of the medical treatment was available for 6 dogs. The medical treatment had somewhat reduced the occurrence of seizures in two dogs, and reduced it by half in two dogs. Two dogs had had no seizures after the introduction of the medication. The medication had reduced the severity of ictal symptoms of only one dog. No side effects were recognised in five dogs (five replied), although the medication was reported to affect the hunting instinct of one dog.

Epilepsy questionnaires were collected from sixteen Beagles in the mainly German subpopulation and fourteen fulfilled our inclusion criteria for IE. The median age of epilepsy onset was 2.5 years, and the dogs had a median of ten seizures per year. Two dogs had experienced more than one seizure within 24 hours. The median seizure duration was three minutes, but the duration ranged between 10 seconds and 5 minutes. Seizure-triggering factors were recognised in only one dog and they were stress and strong emotional status. No typical pre-ictal symptoms were reported. The most typical ictal symptoms were motor, manifesting as tonic-clonic, tonic, clonic movements and tremor of limbs and the whole body. Behavioural symptoms were recognised as seeking out the owner. The dogs also had autonomous symptoms typically manifesting as salivation and urination. The dogs recovered from the seizures in a median of 40 minutes, but for two dogs it took up to 24 hours. Fatigue and confusion were the most typical post-ictal symptoms.

Sixty-four percent (9/14) of the dogs were treated with anti-epileptic medication. Five dogs were treated with phenobarbital monotherapy, one with kaliumbromide monotherapy, one with phenobarbital and kaliumbromide, one with phenobarbital and zonisamide, and one with a combination of phenobarbital, kaliumbromide and gabapentin. The efficacy of the medical treatment was not assessed.

Clinical examination

The clinical examination supported diagnosis of IE in the studied breeds. The Finnish Spitz and Belgian Shepherd examinations have been reported previously [1, 2].

Clinical examinations were performed for eleven epileptic and five healthy Schipperkes. All eleven cases were normal in clinical and neurological examination and six had normal results in the blood tests. Three cases had increased levels of alkaline phosphatase in their blood: this was probably due to the antiepileptic medication in two dogs. The third dog had only mild elevation of alkaline phosphatase. One case had increased levels of glucose in the blood, but the level was normal in the follow up after a couple of days. Cerebrospinal fluid (CSF) was analysed from eight cases, and it was normal. Magnetic resonance imaging (MRI) was performed on 10 cases with no remarkable findings. Electroencephalography (EEG) recordings were performed for eleven cases, but in none of dogs clear epileptic activity was found in visual analysis. As a summary, the dogs' seizures were most likely not caused by the findings in the clinical examinations.

All five controls showed normal clinical and neurological examination. Blood tests were normal in all but one control, who had an increased level of alkaline phosphatase. CSF was analysed from four controls, and it was normal. MRI results were normal in all controls. EEG recordings were performed for five controls, but visual evaluation detected no pathological findings. One of the controls had one seizure episode when she was 15 years of age, approximately five years after the clinical examination was performed.

References

1. Seppala EH, Koskinen LL, Gullov CH, Jokinen P, Karlskov-Mortensen P, Bergamasco L, Baranowska Korberg I, Cizinauskas S, Oberbauer AM, Berendt M, Fredholm M, Lohi H: Identification of a novel idiopathic epilepsy locus in Belgian Shepherd dogs. PLoS One 2012, 7(3):e33549.

2. Viitmaa R, Cizinauskas S, Orro T, Niilo-Rama M, Gordin E, Lohi H, Seppala EH, Bragge H, Snellman M: Phenotype, inheritance characteristics, and risk factors for idiopathic epilepsy in Finnish Spitz dogs. J Am Vet Med Assoc 2013, 243(7):1001-1009.
