# Supplementary material for: Identification of a common risk haplotype for canine idiopathic epilepsy in the ADAM23 gene
Source: BMC Genomics. 2015 Jun 18;16(1):465. doi: 10.1186/s12864-015-1651-9 (PMC4470040; doi:10.1186/s12864-015-1651-9)
Supplement: Additional file 2: Table S1. — The main characteristics of idiopathic epilepsy in four dog breeds based on owner-filled epilepsy questionnaires collected in 2003–2013. [file 12864_2015_1651_MOESM2_ESM.pdf]

Additional Tables:

Additional Table 1. The main characteristics of idiopathic epilepsy in four dog breeds based on owner-filled epilepsy questionnaires collected in 2003-2013.

	Schipperke	Beagle (Finnish hunting)	Beagle (mostly German)	Finnish Spitz (Viitmaa *et al.* 2013)	Belgian Shepherd (Groenendael & Tervueren) (Seppälä *et al.* 2012)
Dogs n	56	16	14	143	94
Males n (castrated)	32 (9)	12	10	86 (5)	42 (21)
Females n (sterilised)	24 (10)	4 (1)	4	57 (3)	52 (21)
Born between years	1998-2006	1996-2006	2000-2006	NA	1982-2010
Mean age of onset years (median; range)	2.9 (1.3; 0.3-5)	2.9 (3; 0.7-5)	2.8 (2.5; 1-5)	median: 3 (range: 7-120 months)	3.3 (3mo-9 years)
Mean number of seizures (median; range)	10 (7; 2-50)	17 (15; 2-30)	21.6 (11; 2-62)	NA	10 (2-100)
Seizures per year (median; range)	7 (4; 1-36)	11 (10; 1-20)	11 (10; 2-24)	median 2 (range 0.5-48)	5 (0,5/year-10/day)
Cluster seizures (more than one seizure in 24h) n (%)	14 (25%)	1 (6%)	2/14 (14%)	22/136 (16.2%)	28% (32)
Mean seizure duration (median, range)	11.2 min (4; 0,5-120)	6.1 min (6; 0.5-20)	3 min (3; 10 s-5 min)	median 10 min (range 1-80 min)	4 min (0,5-60)
Typical triggering factors	Stress (16), sexual activity (4), none (9).	Excitement (4), stress (4), waking up in the morning (3), none (3)	No typical triggering factors	Stress or exercise (79), sleep or rest (35), randomly (49)	Stress, sexual activity
Owner is able to predict the seizure is coming n (%)	17 (34%)	6 (40%)	4/10 (40%)	87/127 (68.6%)	32/74 (43%)
Typical pre-ictal symptoms	Seek out the owner, restlessness, nausea	Seeking contact to the owner, restlessness, nausea	No typical symptoms reported	Hiding without reason, repeated changing of position, licking movements, weakness or tremors, vomiting	Seeking contact to the owner; restlessness; drooling and nausea
Typical ictal symptoms	Stiffening of limbs and neck, muscle fasciculation, tremor, staring	Stiffening of limbs and neck, falling, muscle fasciculation, tremor, staring, trying to get close to the owner.	Stiffening of neck and limbs, muscle fasciculation, tremor, urination, drooling, trying to get near people	Motor signs (n = 123) as tremors, weakness, tonic-clonic or tonic; Autonomic signs (n= 105) as salivation, vomiting or regurgitation or urination;	Stiffening of limbs and neck, muscle fasciculation, tremor, falling, drooling, staring and urination

Typical seizure type	Focal with secondary generalisation	Focal with secondary generalisation	Focal with secondary generalisation	Focal onset (85.1%), Complex focal seizure with secondary generalisation (41.1%)	Focal generalised (37%)
Consciousness during the seizure n (%)					
Normal	4 (12%)	NA	NA	3	NA
Abnormal but not fully absent	26 (79%)	10 (67%)	11 (85%)	56	29 (45%)
Fully absent	3 (9%)	5 (33%)	2 (15%)	67	12 (35%)
The duration and intensity of the seizures has n (%)					
Increased	10 (42%)	2 (22%)	NA	NA	NA
Remained the same	5 (21%)	2 (22%)	NA	not progressing 67.8%	NA
Decreased	9 (38%)	5 (56%)	NA	NA	NA
Typical post-ictal symptoms	Fatigue, confusion	Fatigue, increased drinking	Fatigue, confusion	Restlessness, impaired responsiveness, thirst, and hunger	Fatigue, restlessness
Recovery time after the seizure	70% of the dogs recover in less than 30 minutes.	63% of the dogs recovered in less than 30 minutes	Median: 40 min (range 0 min - 24h)	Median: 21.3 min (range 5-75)	Majority <1 h; many < 15 min, some required hours
Anti-epileptic medication n (%)	18 (32 %)	9 (56%)	9 (64%)	42 (30.7%)	48 (51%)
Effectiveness of the medical treatment n (%)					
Seizures disappeared	2 (15%)	2 (33%)	NA	Treatment was considered to be effective in 30 of 38 (78.9%) dogs	18 (55%)
Seizures reduced by half	4 (31%)	2 (33%)	NA		10 (30%)
Seizures somewhat reduced	4 (31%)	2 (33%)	NA		4 (12%)
No effects	3 (23%)	-	NA		1 (3%)
