# Supplementary material for: Identification of a common risk haplotype for canine idiopathic epilepsy in the ADAM23 gene
Source: BMC Genomics. 2015 Jun 18;16(1):465. doi: 10.1186/s12864-015-1651-9 (PMC4470040; doi:10.1186/s12864-015-1651-9)
Supplement: Additional file 3: Figure S1. — Multidimensional scaling plots. [file 12864_2015_1651_MOESM3_ESM.pdf]

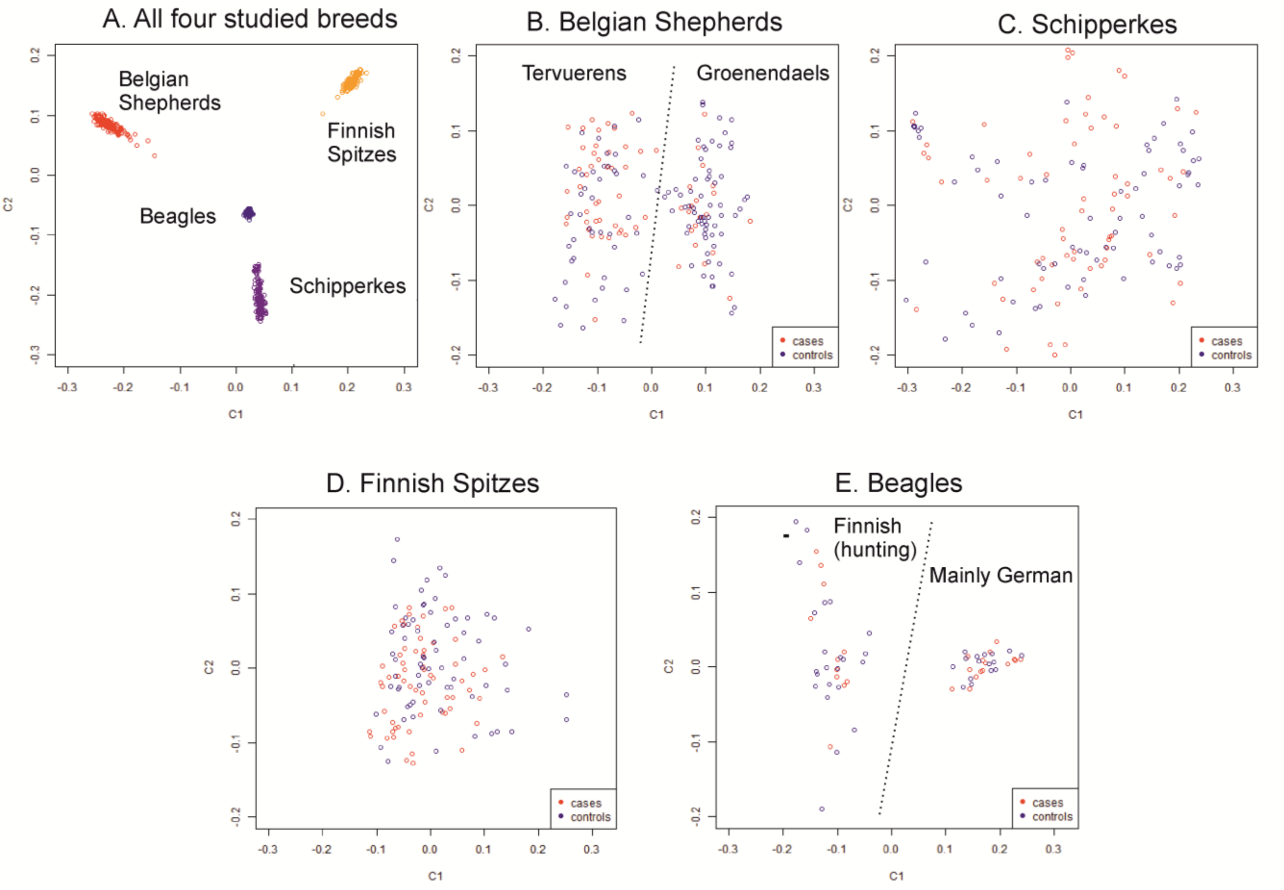

Additional Figure 1. Multidimensional scaling plots. All studied breeds (**A**) were distinct genetic populations, presented by multidimensional scaling plots. There is no substructure in the Finnish Spitz (**D**) and Schipperke (**C**) cohorts. In Belgian Shepherd (**B**) and Beagle (**E**) cohorts, two subpopulations were identified, and represent breed lines.
