# Supplementary material for: Identification of a common risk haplotype for canine idiopathic epilepsy in the ADAM23 gene
Source: BMC Genomics. 2015 Jun 18;16(1):465. doi: 10.1186/s12864-015-1651-9 (PMC4470040; doi:10.1186/s12864-015-1651-9)
Supplement: Additional file 4: Figure S2. — Results of the genome-wide association study (GWAS). [file 12864_2015_1651_MOESM4_ESM.pdf]

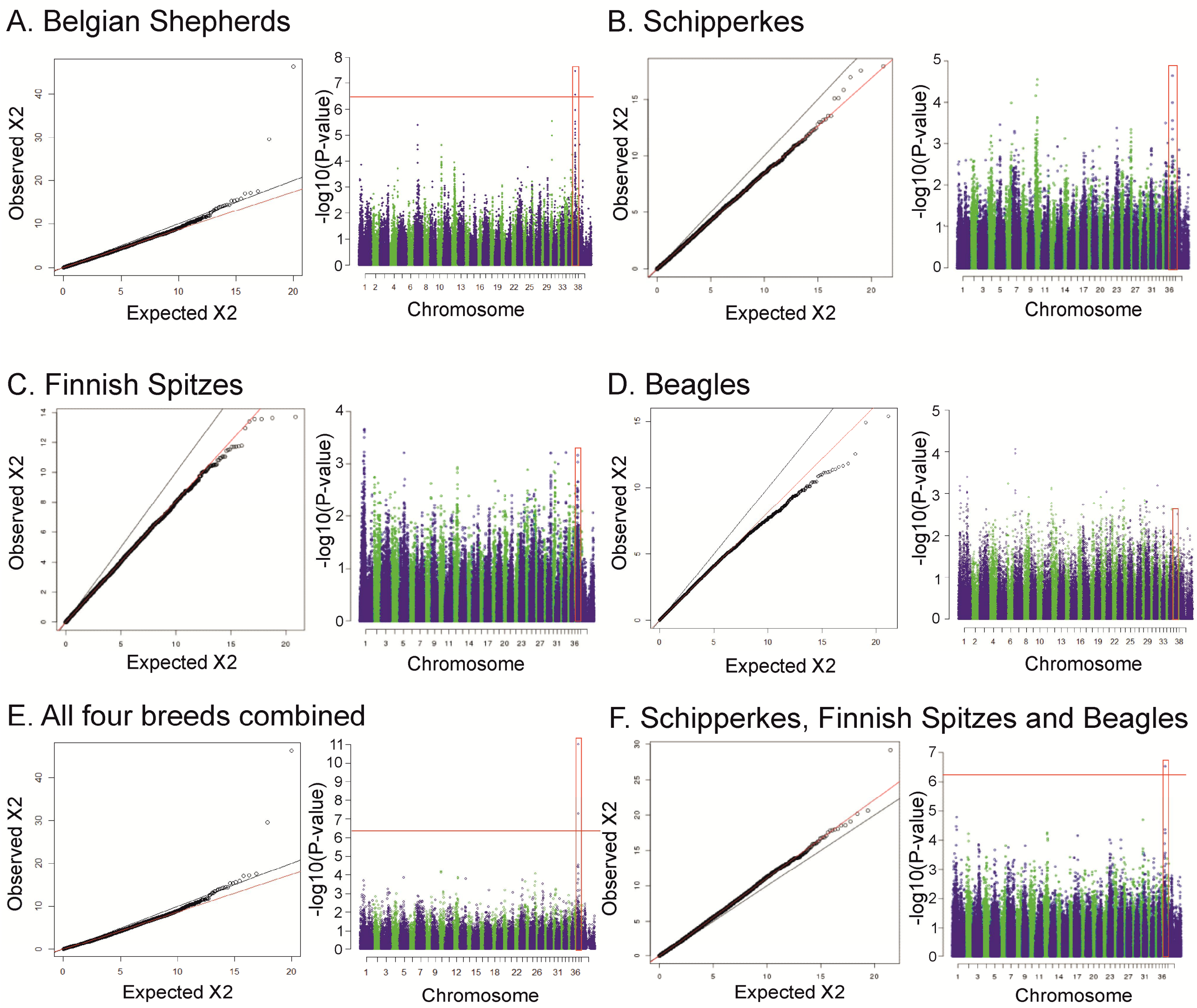

Additional Figure 2. Results of the genome-wide association study (GWAS). Quantile-quantile and association plots of the GWAS in four dog breeds individually (A-D), and combined cohorts (E-F). The red line on the Manhattan plots for Belgian Shepherds (A), Schipperkes, Finnish Spitzs and Beagles (E) and all four breeds (F) represents the p-value level of genome-wide significance. The GWAS in Schipperkes, Finnish Spitzs or Beagles alone did not reach genome-wide significance. CFA37 is highlighted with a red outline in the Manhattan plots.
