# Supplementary material for: Identification of a common risk haplotype for canine idiopathic epilepsy in the ADAM23 gene
Source: BMC Genomics. 2015 Jun 18;16(1):465. doi: 10.1186/s12864-015-1651-9 (PMC4470040; doi:10.1186/s12864-015-1651-9)
Supplement: Additional file 5: Figure S3. — The Schipperke pedigree. [file 12864_2015_1651_MOESM5_ESM.pdf]

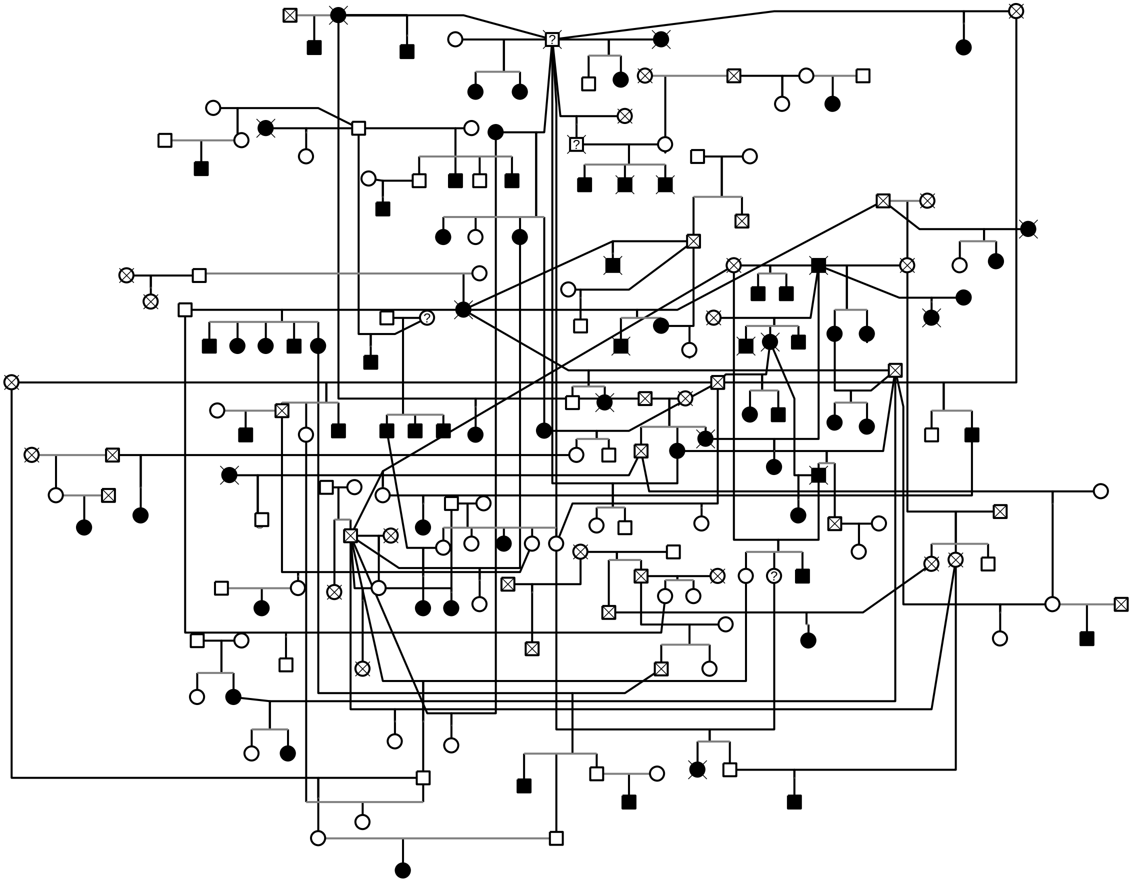

Additional Figure 3. The Schipperke pedigree. The Schipperke cohort consisted mainly of closely related individuals, which could be drawn on the same pedigree consisting of multiple epilepsy cases (indicated by black circles and squares) and healthy control dogs (indicated by white circles and squares). Circle: female; square: male; X: deceased
