# Supplementary material for: Identification of a common risk haplotype for canine idiopathic epilepsy in the ADAM23 gene
Source: BMC Genomics. 2015 Jun 18;16(1):465. doi: 10.1186/s12864-015-1651-9 (PMC4470040; doi:10.1186/s12864-015-1651-9)
Supplement: Additional file 6: Table S2. — A summary of the results of the resequencing of a 4.4 Mb locus on CFA37 in 12 Belgian Shepherd cases and 12 controls with epilepsy. [file 12864_2015_1651_MOESM6_ESM.pdf]

Additional Table 2. A summary of the results of the resequencing of a 4.4 Mb locus on CFA37 in 12 Belgian Shepherd cases and 12 controls with epilepsy, homozygous with respect to opposite haplotypes based on the previous GWAS results. CanFam3.1 was used as a reference sequence.

					Total number of variants		Number of variants in coding regions	
		**No of reads**	**% mapped[a]**	**Coverage[b]**	**SNVs**	**indels**	**SNVs**	**indels**
**Case #**	**1**	10151606	56.15	119.98	7263	3643	49	9
	**2**	8502512	51.5	92.16	9406	4096	55	9
	**3**	9419516	50.92	100.94	7289	3599	52	9
	**4**	10523428	47.12	104.45	9169	3982	66	10
	**5**	9460132	49.17	97.94	7629	3749	50	9
	**6**	7270666	50.04	76.55	7133	3560	47	9
	**7**	8403172	47.62	84.19	9014	3822	59	10
	**8**	9312952	50	98.00	8137	3909	61	10
	**9**	8652570	55.11	100.38	9463	4092	58	10
	**10**	9051446	52.04	99.15	9433	4206	61	10
	**11**	8199798	46.65	80.48	8012	3884	51	9
	**12**	6520976	53.11	72.88	8664	3829	63	9
**Control #**	**1**	9932028	53.02	110.86	9155	4120	63	11
	**2**	8043084	53.36	90.34	5934	2922	49	9
	**3**	8872118	53.86	100.62	6981	3306	54	10
	**4**	8878946	52.7	98.54	7174	3333	51	11
	**5**	7656894	50.48	81.365	7979	3545	53	9
	**6**	8590408	46.24	83.62	6238	3061	51	9
	**7**	11380260	43	103.11	9009	3973	63	10
	**8**	8088498	50.35	85.73	8019	3600	55	10
	**9**	7244644	54.22	82.68	7884	3481	53	9
	**10**	7171088	50.73	76.57	7983	3564	59	9
	**11**	5503566	46.83	54.24	7985	3394	48	9
	**12**	7742696	54.14	88.25	5946	2922	49	9

[a] Percentage of reads aligned to target region after duplicate removal

[b] Average sequencing coverage on target

SNV: single nucleotide variant; indel: insertion-deletion variant
