# Supplementary material for: Identification of a common risk haplotype for canine idiopathic epilepsy in the ADAM23 gene
Source: BMC Genomics. 2015 Jun 18;16(1):465. doi: 10.1186/s12864-015-1651-9 (PMC4470040; doi:10.1186/s12864-015-1651-9)
Supplement: Additional file 7: Table S3. — Association results of the validation of the 26 ADAM23 variants in 285 cases and 355 controls with idiopathic epilepsy. [file 12864_2015_1651_MOESM7_ESM.pdf]

Additional Table 3. Association results of the validation of the 26 *ADAM23* variants in 285 cases and 355 controls with idiopathic epilepsy.

CHR37_15085438 (BICF2S23030950); INTRONIC TO *ADAM23*						P(CMH) 1.3e-14	OR 3.043 (2.278-4.063)			
Breed	A1	F_A	F_U	A2	CHISQ	P	OR	SE	L95	U95
Belgian Shepherds	*T*	0.756	0.4766	*G*	46.77	7.985e-12	3.402	0.1828	2.378	4.868
Schipperkes	*T*	0.9706	0.9928	*G*	1.878	0.1705	0.2409	1.125	0.02658	2.183
Finnish Spitzs	*T*	0.8898	0.6716	*G*	17.07	3.609e-05	3.949	0.3468	2.001	7.792
Beagles (Finnish hunting)	*T*	0.9643	0.7292	*G*	6.505	0.01076	10.03	1.069	1.234	81.49
Beagles (other)	*T*	0.625	0.6154	*G*	0.004897	0.9442	1.042	0.5833	0.332	3.268
CHR37_15093174 (BICF2P1290526); INTRONIC TO *ADAM23*						P(CMH) 4.121e-06	OR 0.5563 (0.4329-0.7149)			
Breed	A1	F_A	F_U	A2	CHISQ	P	OR	SE	L95	U95
Belgian Shepherds	*T*	0.7024	0.7386	*C*	0.965	0.3259	0.8351	0.1836	0.5827	1.197
Schipperkes	*T*	0.1866	0.3714	*C*	11.58	0.0006666	0.3881	0.2824	0.2231	0.6752
Finnish Spitzs	*T*	0.123	0.3603	*C*	19.42	1.048e-05	0.2489	0.3285	0.1307	0.4739
Beagles (Finnish hunting)	*T*	0.03571	0.32	*C*	8.51	0.003531	0.0787	1.063	0.009808	0.6316
Beagles (other)	*T*	0.4667	0.4062	*C*	0.2299	0.6316	1.279	0.5133	0.4676	3.497
CHR37_15106446 (BICF2P1021781); INTRONIC TO *ADAM23*						P(CMH) 5.297e-15	OR 2.878 (2.197-3.77)			
Breed	A1	F_A	F_U	A2	CHISQ	P	OR	SE	L95	U95
Belgian Shepherds	*C*	0.756	0.4794	*T*	45.8	1.313e-11	3.364	0.1829	2.351	4.815
Schipperkes	*C*	0.8088	0.65	*T*	8.793	0.003024	2.278	0.281	1.313	3.951
Finnish Spitzs	*C*	0.9576	0.8582	*T*	7.197	0.007301	3.734	0.5198	1.348	10.34
Beagles (Finnish hunting)	*C*	0.9643	0.7083	*T*	7.313	0.006845	11.12	1.067	1.374	89.95
Beagles (other)	*C*	0.6333	0.6333	*T*	0	1	1	0.5358	0.3499	2.858
CHR37_15108593; INTRONIC TO *ADAM23*						P(CMH) 5.052e-15	OR 2.865 (2.19-3.748)			
Breed	A1	F_A	F_U	A2	CHISQ	P	OR	SE	L95	U95
Belgian Shepherds	*del*	0.752	0.4766	*A*	45.82	1.294e-11	3.329	0.1812	2.334	4.749
Schipperkes	*del*	0.8088	0.65	*A*	8.793	0.003024	2.278	0.281	1.313	3.951
Finnish Spitzs	*del*	0.9576	0.8582	*A*	7.197	0.007301	3.734	0.5198	1.348	10.34
Beagles (Finnish hunting)	*del*	0.9643	0.7083	*A*	7.313	0.006845	11.12	1.067	1.374	89.95
Beagles (other)	*del*	0.6333	0.6333	*A*	0	1	1	0.5358	0.3499	2.858
CHR37_15108802; INTRONIC TO *ADAM23*						P(CMH) 5.052e-15	OR 2.865 (2.19-3.748)		2.19	3.748
Breed	A1	F_A	F_U	A2	CHISQ	P	OR	SE	L95	U95

Belgian Shepherds	*del*	0.752	0.4766	*T*	45.82	1.294e-11	3.329	0.1812	2.334	4.749
Schipperkes	*del*	0.8088	0.65	*T*	8.793	0.003024	2.278	0.281	1.313	3.951
Finnish Spitzs	*del*	0.9576	0.8582	*T*	7.197	0.007301	3.734	0.5198	1.348	10.34
Beagles (Finnish hunting)	*del*	0.9643	0.7083	*T*	7.313	0.006845	11.12	1.067	1.374	89.95
Beagles (other)	*del*	0.6333	0.6333	*T*	0	1	1	0.5358	0.3499	2.858
CHR37_15111724 (BICF2P1131874); INTRONIC TO *ADAM23*						P(CMH) 4.15e-15	OR 2.892 (2.207-3.79)			
Breed	A1	F_A	F_U	A2	CHISQ	P	OR	SE	L95	U95
Belgian Shepherds	*G*	0.756	0.4856	*A*	44.26	2.872e-11	3.282	0.1822	2.296	4.69
Schipperkes	*G*	0.8209	0.65	*A*	10.23	0.001381	2.468	0.2866	1.407	4.328
Finnish Spitzs	*G*	0.959	0.8529	*A*	8.269	0.004033	4.034	0.5169	1.465	11.11
Beagles (Finnish hunting)	*G*	0.9643	0.72	*A*	6.896	0.00864	10.5	1.066	1.3	84.83
Beagles (other)	*G*	0.6333	0.6562	*A*	0.03552	0.8505	0.9048	0.5311	0.3195	2.562
CHR37_15113325; INTRONIC TO *ADAM23*						P(CMH) 2.757e-15	OR 2.932 (2.234-3.849)			
Breed	A1	F_A	F_U	A2	CHISQ	P	OR	SE	L95	U95
Belgian Shepherds	*G*	0.754	0.4766	*A*	46.29	1.019e-11	3.365	0.182	2.356	4.808
Schipperkes	*G*	0.8088	0.65	*A*	8.793	0.003024	2.278	0.281	1.313	3.951
Finnish Spitzs	*G*	0.9737	0.8692	*A*	8.807	0.003002	5.566	0.6403	1.587	19.53
Beagles (Finnish hunting)	*G*	0.9643	0.7174	*A*	6.917	0.00854	10.64	1.07	1.307	86.56
Beagles (other)	*G*	0.6333	0.6333	*A*	0	1	1	0.5358	0.3499	2.858
CHR37_15113726; INTRONIC TO *ADAM23*						P(CMH) 1.326e-10	OR 2.499 (1.882-3.318)			
Breed	A1	F_A	F_U	A2	CHISQ	P	OR	SE	L95	U95
Belgian Shepherds	*G*	0.7619	0.4688	*A*	26.93	2.112e-07	3.627	0.2543	2.203	5.97
Schipperkes	*G*	0.8308	0.6167	*A*	14.45	0.0001443	3.052	0.2999	1.695	5.493
Finnish Spitzs	*G*	0.7105	0.5909	*A*	3.829	0.05037	1.699	0.272	0.9971	2.896
Beagles (Finnish hunting)	*G*	0.9643	0.6667	*A*	9.021	0.00267	13.5	1.063	1.679	108.5
Beagles (other)	*G*	0.5333	0.5938	*A*	0.2299	0.6316	0.782	0.5133	0.2859	2.138
CHR37_15113939; *ADAM23* EXON12; *c. 1158C>T*, p.R387C						P(CMH) 0.001441	OR 0.5887 (0.4247-0.8159)			
Breed	A1	F_A	F_U	A2	CHISQ	P	OR	SE	L95	U95
Belgian Shepherds	*C*	0.9937	1	*T*	1.637	0.2008	0	Inf	0	nan
Schipperkes	*C*	0.4179	0.587	*T*	7.772	0.005307	0.5052	0.2461	0.3119	0.8184
Finnish Spitzs	*C*	0.3534	0.4776	*T*	3.935	0.04729	0.5979	0.2601	0.3591	0.9954
Beagles (Finnish hunting)	*C*	0.8929	0.8913	*T*	0.0004353	0.9834	1.016	0.7731	0.2233	4.625

Beagles (other)	*C*	0.8333	0.7812	*T*	0.2691	0.6039	1.4	0.6503	0.3914	5.008
CHR37_15113940, *ADAM23* EXON12; *c.1159G>A*, p.R387H						P(CMH) 1.671e-08	OR 2.098 (1.617-2.722)			
Breed	A1	F_A	F_U	A2	CHISQ	P	OR	SE	L95	U95
Belgian Shepherds	*A*	0.748	0.4766	*G*	44.47	2.589e-11	3.26	0.1806	2.288	4.645
Schipperkes	*A*	0.2164	0.2174	*G*	0.0003792	0.9845	0.9943	0.2943	0.5585	1.77
Finnish Spitzs	*A*	0.0678	0.06716	*G*	0.0003989	0.9841	1.01	0.5032	0.3767	2.708
Beagles (Finnish hunting)	*A*	0.8571	0.56	*G*	7.131	0.007574	4.714	0.6106	1.425	15.6
Beagles (other)	*A*	0.3667	0.375	*G*	0.004608	0.9459	0.9649	0.5262	0.344	2.706
CHR37_15114127; INTRONIC TO *ADAM23*						P(CMH) 1.17e-11	OR 2.506 (1.915-3.28)			
Breed	A1	F_A	F_U	A2	CHISQ	P	OR	SE	L95	U95
Belgian Shepherds	*T*	0.7595	0.469	*G*	33.93	5.707e-09	3.575	0.2241	2.305	5.547
Schipperkes	*T*	0.8154	0.627	*G*	11.33	0.000762	2.628	0.2916	1.484	4.654
Finnish Spitzs	*T*	0.7155	0.5896	*G*	4.325	0.03755	1.751	0.2705	1.03	2.976
Beagles (Finnish hunting)	*T*	0.9643	0.6667	*G*	9.021	0.00267	13.5	1.063	1.679	108.5
Beagles (other)	*T*	0.5333	0.5938	*G*	0.2299	0.6316	0.782	0.5133	0.2859	2.138
CHR37_15114156; INTRONIC TO *ADAM23*						P(CMH) 2.686e-14	OR 2.559 (2.002-3.271)			
Breed	A1	F_A	F_U	A2	CHISQ	P	OR	SE	L95	U95
Belgian Shepherds	*C*	0.7602	0.4763	*T*	47.63	5.144e-12	3.484	0.1848	2.426	5.006
Schipperkes	*C*	0.8015	0.6286	*T*	10.09	0.001487	2.386	0.2771	1.386	4.107
Finnish Spitzs	*C*	0.7203	0.5896	*T*	4.723	0.02977	1.793	0.27	1.056	3.044
Beagles (Finnish hunting)	*C*	0.9643	0.68	*T*	8.51	0.003531	12.71	1.063	1.583	102
Beagles (other)	*C*	0.5333	0.5938	*T*	0.2299	0.6316	0.782	0.5133	0.2859	2.138
CHR37_15114233; INTRONIC TO *ADAM23*						P(CMH) 9.329e-05	OR 1.83 (1.348-2.484)			
Breed	A1	F_A	F_U	A2	CHISQ	P	OR	SE	L95	U95
Belgian Shepherds	*T*	0.7578	0.4796	*C*	24.77	6.464e-07	3.395	0.251	2.076	5.553
Schipperkes	*T*	0.2273	0.2266	*C*	0.0001869	0.9891	1.004	0.2962	0.5619	1.794
Finnish Spitzs	*T*	0.06897	0.06716	*C*	0.003183	0.955	1.029	0.5034	0.3836	2.759
Beagles (Finnish hunting)	*T*	0.8462	0.5417	*C*	6.861	0.008811	4.654	0.6159	1.392	15.56
Beagles (other)	*T*	0.3667	0.375	*C*	0.004608	0.9459	0.9649	0.5262	0.344	2.706
CHR37_15114321; INTRONIC TO *ADAM23*						P(CMH) 5.074e-07	OR 2.088 (1.563-2.788)			
Breed	A1	F_A	F_U	A2	CHISQ	P	OR	SE	L95	U95
Belgian Shepherds	*T*	0.6842	0.4635	*C*	10.63	0.00111	2.507	0.2861	1.431	4.393

Schipperkes	*T*	0.8	0.629	*C*	9.133	0.00251	2.359	0.2875	1.343	4.144
Finnish Spitzs	*T*	0.6696	0.5299	*C*	4.942	0.02622	1.799	0.2652	1.07	3.025
Beagles (Finnish hunting)	*T*	0.9286	0.625	*C*	8.405	0.003743	7.8	0.7921	1.652	36.84
Beagles (other)	*T*	0.5333	0.5938	*C*	0.2299	0.6316	0.782	0.5133	0.2859	2.138
CHR37_15114336; INTRONIC TO *ADAM23*						P(CMH) 3.5e-12	OR 2.587 (1.971-3.395)			
Breed	A1	F_A	F_U	A2	CHISQ	P	OR	SE	L95	U95
Belgian Shepherds	*C*	0.7628	0.4685	*G*	34.38	4.541e-09	3.649	0.2264	2.341	5.686
Schipperkes	*C*	0.8385	0.6349	*G*	13.72	0.0002123	2.985	0.3017	1.652	5.391
Finnish Spitzs	*C*	0.7155	0.5896	*G*	4.325	0.03755	1.751	0.2705	1.03	2.976
Beagles (Finnish hunting)	*C*	0.9643	0.6667	*G*	9.021	0.00267	13.5	1.063	1.679	108.5
Beagles (other)	*C*	0.5333	0.5938	*G*	0.2299	0.6316	0.782	0.5133	0.2859	2.138
CHR37_15115295 (BICF2P968072); INTRONIC TO *ADAM23*						P(CMH) 3.283e-14	OR 2.545 (1.993-3.251)			
Breed	A1	F_A	F_U	A2	CHISQ	P	OR	SE	L95	U95
Belgian Shepherds	*A*	0.756	0.4766	*G*	46.77	7.985e-12	3.402	0.1828	2.378	4.868
Schipperkes	*A*	0.8015	0.6286	*G*	10.09	0.001487	2.386	0.2771	1.386	4.107
Finnish Spitzs	*A*	0.7203	0.5909	*G*	4.6	0.03197	1.783	0.2709	1.049	3.033
Beagles (Finnish hunting)	*A*	0.9643	0.6875	*G*	8.151	0.004303	12.27	1.065	1.522	98.94
Beagles (other)	*A*	0.5333	0.5667	*G*	0.06734	0.7952	0.8739	0.5193	0.3158	2.418
CHR37_15115366 (BICF2P968073); INTRONIC TO *ADAM23*						P(CMH) 3.594e-14	OR 2.536 (1.987-3.237)			
Breed	A1	F_A	F_U	A2	CHISQ	P	OR	SE	L95	U95
Belgian Shepherds	*A*	0.754	0.4766	*G*	46.29	1.019e-11	3.365	0.182	2.356	4.808
Schipperkes	*A*	0.8015	0.6286	*G*	10.09	0.001487	2.386	0.2771	1.386	4.107
Finnish Spitzs	*A*	0.7203	0.5896	*G*	4.723	0.02977	1.793	0.27	1.056	3.044
Beagles (Finnish hunting)	*A*	0.9643	0.6875	*G*	8.151	0.004303	12.27	1.065	1.522	98.94
Beagles (other)	*A*	0.5333	0.5667	*G*	0.06734	0.7952	0.8739	0.5193	0.3158	2.418
CHR37_15116559; INTRONIC TO *ADAM23*						P(CMH) 4.493e-12	OR 2.404 (1.868-3.095)			
Breed	A1	F_A	F_U	A2	CHISQ	P	OR	SE	L95	U95
Belgian Shepherds	*C*	0.7602	0.4766	*T*	47.75	4.844e-12	3.481	0.1844	2.425	4.996
Schipperkes	*C*	0.8088	0.65	*T*	8.793	0.003024	2.278	0.281	1.313	3.951
Finnish Spitzs	*C*	0.7881	0.7761	*T*	0.0531	0.8178	1.073	0.3061	0.5889	1.955
Beagles (Finnish hunting)	*C*	0.9643	0.7083	*T*	7.313	0.006845	11.12	1.067	1.374	89.95
Beagles (other)	*C*	0.6333	0.6333	*T*	0	1	1	0.5358	0.3499	2.858

CHR37_15118894; INTRONIC TO *ADAM23*						P(CMH) 1.138e-11	OR 2.334 (1.82-2.991)			
Breed	A1	F_A	F_U	A2	CHISQ	P	OR	SE	L95	U95
Belgian Shepherds	*G*	0.752	0.4794	*A*	44.85	2.123e-11	3.292	0.1814	2.307	4.697
Schipperkes	*G*	0.8015	0.6286	*A*	10.09	0.001487	2.386	0.2771	1.386	4.107
Finnish Spitzs	*G*	0.7881	0.7761	*A*	0.0531	0.8178	1.073	0.3061	0.5889	1.955
Beagles (Finnish hunting)	*G*	0.9643	0.6875	*A*	8.151	0.004303	12.27	1.065	1.522	98.94
Beagles (other)	*G*	0.5333	0.5667	*A*	0.06734	0.7952	0.8739	0.5193	0.3158	2.418
CHR37_15119387; INTRONIC TO *ADAM23*						P(CMH) 4.151e-15	OR 2.881 (2.201-3.771)			
Breed	A1	F_A	F_U	A2	CHISQ	P	OR	SE	L95	U95
Belgian Shepherds	C	0.754	0.4766	T	46.29	1.019e-11	3.365	0.182	2.356	4.808
Schipperkes	C	0.8088	0.65	T	8.793	0.003024	2.278	0.281	1.313	3.951
Finnish Spitzs	C	0.9576	0.8582	T	7.197	0.007301	3.734	0.5198	1.348	10.34
Beagles (Finnish hunting)	C	0.9643	0.7083	T	7.313	0.006845	11.12	1.067	1.374	89.95
Beagles (other)	C	0.6333	0.6333	T	0	1	1	0.5358	0.3499	2.858
CHR37_15121756 (BICF2P86747); INTRONIC TO *ADAM23*						P(CMH) 1.686e-10	OR 2.726 (1.993-3.729)			
Breed	A1	F_A	F_U	A2	CHISQ	P	OR	SE	L95	U95
Belgian Shepherds	*C*	0.7619	0.4912	*T*	44.52	2.512e-11	3.314	0.1832	2.314	4.746
Schipperkes	*C*	0.9485	0.9643	*T*	0.4118	0.5211	0.6825	0.5983	0.2113	2.205
Finnish Spitzs	*C*	1	0.9925	*T*	0.8841	0.3471	NA	NA	NA	NA
Beagles (Finnish hunting)	*C*	0.9643	0.75	*T*	5.727	0.01671	9	1.072	1.102	73.51
Beagles (other)	*C*	0.7	0.6667	*T*	0.07702	0.7814	1.167	0.5556	0.3926	3.467
CHR37_15121845; INTRONIC TO *ADAM23*						P(CMH) 3.682e-14	OR 2.705 (2.081-3.514)			
Breed	A1	F_A	F_U	A2	CHISQ	P	OR	SE	L95	U95
Belgian Shepherds	*del*	0.754	0.4794	*T*	45.32	1.673e-11	3.328	0.1821	2.329	4.755
Schipperkes	*del*	0.8015	0.6286	*T*	10.09	0.001487	2.386	0.2771	1.386	4.107
Finnish Spitzs	*del*	0.9237	0.8433	*T*	3.872	0.0491	2.251	0.4204	0.9873	5.131
Beagles (Finnish hunting)	*del*	0.9643	0.6875	*T*	8.151	0.004303	12.27	1.065	1.522	98.94
Beagles (other)	*del*	0.5333	0.5667	*T*	0.06734	0.7952	0.8739	0.5193	0.3158	2.418
CHR37_15123005 (BICF2P86749); INTRONIC TO *ADAM23*						P(CMH) 1.452e-10	OR 2.789 (2.027-3.838)			
Breed	A1	F_A	F_U	A2	CHISQ	P	OR	SE	L95	U95
Belgian Shepherds	*C*	0.7598	0.4912	*A*	44.03	3.227e-11	3.277	0.1824	2.292	4.685
Schipperkes	*C*	0.9706	0.9929	*A*	1.923	0.1655	0.2374	1.125	0.0262	2.152

Finnish Spitzs	*C*	1	0.9925	*A*	0.8841	0.3471	NA	NA	NA	NA
Beagles (Finnish hunting)	*C*	0.9643	0.75	*A*	5.727	0.01671	9	1.072	1.102	73.51
Beagles (other)	*C*	0.7	0.6667	*A*	0.07702	0.7814	1.167	0.5556	0.3926	3.467
CHR37_15123950; INTRONIC TO *ADAM23*						P(CMH) 9.56e-11	OR 2.835 (2.056-3.91)			
Breed	A1	F_A	F_U	A2	CHISQ	P	OR	SE	L95	U95
Belgian Shepherds	*AA*	0.764	0.4912	*del*	45.02	1.947e-11	3.353	0.1841	2.337	4.81
Schipperkes	*AA*	0.9706	0.9929	*del*	1.923	0.1655	0.2374	1.125	0.0262	2.152
Finnish Spitzs	*AA*	1	0.9925	*del*	0.8841	0.3471	NA	NA	NA	NA
Beagles (Finnish hunting)	*AA*	0.9643	0.75	*del*	5.727	0.01671	9	1.072	1.102	73.51
Beagles (other)	*AA*	0.7	0.6667	*del*	0.07702	0.7814	1.167	0.5556	0.3926	3.467
CFA37_15124213 (BICF2P890779); INTRONIC TO *ADAM23*						P(CMH) 5.598e-10	OR 2.687 (1.956-3.693)			
Breed	A1	F_A	F_U	A2	CHISQ	P	OR	SE	L95	U95
Belgian Shepherds	*A*	0.7598	0.5	*G*	41.83	9.953e-11	3.164	0.1815	2.217	4.516
Schipperkes	*A*	0.9706	0.9929	*G*	1.923	0.1655	0.2374	1.125	0.0262	2.152
Finnish Spitzs	*A*	1	0.9925	*G*	0.8841	0.3471	NA	NA	NA	NA
Beagles (Finnish hunting)	*A*	0.9643	0.76	*G*	5.393	0.02022	8.526	1.071	1.045	69.54
Beagles (other)	*A*	0.7	0.6875	*G*	0.01138	0.915	1.061	0.5515	0.3598	3.126
CHR37_15124474 (BICF2P890781); INTRONIC TO *ADAM23*						P(CMH) 1.452e-10	OR 2.789 (2.027-3.838)			
Breed	A1	F_A	F_U	A2	CHISQ	P	OR	SE	L95	U95
Belgian Shepherds	*C*	0.7598	0.4912	*T*	44.03	3.227e-11	3.277	0.1824	2.292	4.685
Schipperkes	*C*	0.9706	0.9929	*T*	1.923	0.1655	0.2374	1.125	0.0262	2.152
Finnish Spitzs	*C*	1	0.9925	*T*	0.8841	0.3471	NA	NA	NA	NA
Beagles (Finnish hunting)	*C*	0.9643	0.75	*T*	5.727	0.01671	9	1.072	1.102	73.51
Beagles (other)	*C*	0.7	0.6667	*T*	0.07702	0.7814	1.167	0.5556	0.3926	3.467
