# Supplementary material for: Identification of a common risk haplotype for canine idiopathic epilepsy in the ADAM23 gene
Source: BMC Genomics. 2015 Jun 18;16(1):465. doi: 10.1186/s12864-015-1651-9 (PMC4470040; doi:10.1186/s12864-015-1651-9)
Supplement: Additional file 8: Figure S4. — Overview of the ADAM23 5’- region and DNA sequence. [file 12864_2015_1651_MOESM8_ESM.pdf]

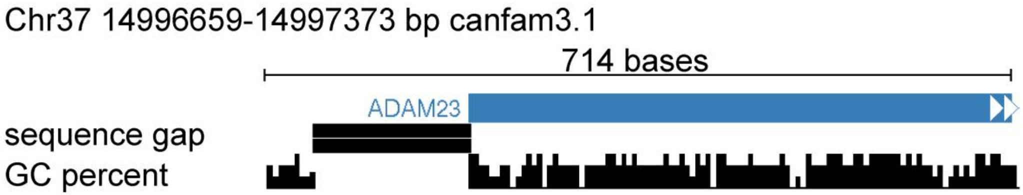

ADAM23 5'
190 bp gap region

CCCTTGCCTCCGCGGCGACGCATGCGCAGTGCGCCCCGTGCCCGGCACT
AGCCACAGCGTCGGGACCGGCGTCGGCGGCAGCATCAGGACCCG**CGGGC**
GCGCTCTCGAGGGCGGGGCGCCCCCCCCCCCCGCCGCGCGCCCCCGCG
CCCCGCGCCCCGCGCCCCGCGCCCCCGCGCCGCGCGCTCGCCCGCGCCG
TCTCAGCATCCTCAGCCCCGGCGGCAGCCCCCGCAGCTGCTGGAGCGGC
CGCGCCCGCCGCAGGGAGGGAGGAGCATCTGGGGCGGCTCCAAGTTGGC
GGAGCCGCCGGGACCCCCGGACTCCTCCGCGGCCGCCCGGGAGGGGCGG
AGAGGCGAGAGGCGAGCAGGGGAGGGGAGCCGCCGGCCGCCGGTGCCGC
GCCCCGAG**CCCCTGCCCCTGCCCCTGCCCCTGCCCCTGCCCCTGCCCCT**
GCCCGCGGCCGCACCATGCGCGCCCAGCCGGCGTGATCGGCCCCGCCCG
CAGCCTCCCCGCAGCCTCCCCCGGCGCTGTCGCCGGCCACACCGAGCGG
CGCCCGGGAGCTATGAGCC**ATG**AAGCCGCCCGGCAGCAGCTCCCGGCGG
CCGCCCCGCGCGGGCTCCAGCCGGCCCCGCGCCTCCCGCGGCCCCCGCG
GCGGCCCCGCCGGCCCGGTGCCCGCCCGCACGCCGCCCTGCCGCCTGCT
CCTCGTCCTTCTCCTGCTGCCTCCTCTCGCCACCTCGTCGCGGCCCCGC
GCCTGGGGGGCGGCTGCGCCCAGCG

ADAM23 exon 1

Additional Figure 4.Overview of the *ADAM23* 5’- region and DNA sequence. Canine BAC clone CHORI82_R4_266G10 spanning the ADAM23 region was sequenced using PacBio sequencing to uncover the genomic sequence of the unknown sequence 5’ of *ADAM23*. The newly-identified 190-bp sequence is shown here along with *ADAM23* exon 1 genomic sequence. The translation starts at ATG highlighted in red. Both the newly-identified sequence and exon 1 have GC content above 80 percent and contain simple repetitive elements, highlighted in bold.
