# Supplementary material for: Identification of a common risk haplotype for canine idiopathic epilepsy in the ADAM23 gene
Source: BMC Genomics. 2015 Jun 18;16(1):465. doi: 10.1186/s12864-015-1651-9 (PMC4470040; doi:10.1186/s12864-015-1651-9)
Supplement: Additional file 9: Table S4. — Variants belonging to the epilepsy risk haplotype in Belgian Shepherds identified in the targeted next-generation sequencing. [file 12864_2015_1651_MOESM9_ESM.pdf]

Additional Table 4. Variants belonging to the IE-risk haplotype in Belgian Shepherds (BS) identified in the targeted next-generation sequencing are listed in black. Variants not identified in the resequencing but included in the validation are highlighted in grey. Twenty-seven variants were included in the validation in BS, Schipperkes, Finnish Spitzs and Beagles. Twenty-five variants were selected based on the targeted resequencing of BS. BICF2P1290526 was included in the validation, as it showed the strongest association in the combined genome-wide association analysis without BS. The non-synonymous variant at 15113939 bp is rare in BS, but was included because it was common in other breeds based on previous studies. The six variants belonging to the shared risk haplotype across breeds are bolded.

Bp on CFA37	Ref.	Alt.	SNP ID	Gene	Position	Function	Validation
15085438	*G*	*T*	BICF2S23030950	*ADAM23*	Intronic	NA	Sequenom
15093174	*T*	*C*	BICF2P1290526*	*ADAM23*	Intronic	NA	Sequenom
15106446	*T*	*C*	BICF2P1021781	*ADAM23*	Intronic	NA	Sequenom
15108133	*T*	*A*	NA	*ADAM23*	intronic	NA	-
15108582	*AA*	-	NA	*ADAM23*	intronic	NA	-
15108593	*A*	-	NA	*ADAM23*	intronic	NA	Sequenom
15108802	*T*	-	NA	*ADAM23*	intronic	NA	Sequenom
15111179	*A*	-	NA	*ADAM23*	intronic	NA	-
15111724	*A*	*G*	BICF2P1131874*	*ADAM23*	intronic	NA	Sequenom
15113325	*A*	*G*	NA	*ADAM23*	intronic	NA	Sequenom
15113726	*A*	*G*	NA	*ADAM23*	intronic	NA	Sangerseq.
15113939	*C*	*T*	NA	*ADAM23*	exonic	nonsynonymous SNV	Sangerseq.
15113940	*G*	*A*	NA	*ADAM23*	exonic	nonsynonymous SNV	Sequenom
15114127	*G*	*T*	NA	*ADAM23*	intronic	NA	Sangerseq.
15114156	*T*	*C*	NA	*ADAM23*	intronic	NA	Sequenom
15114233	*C*	*T*	NA	*ADAM23*	intronic	NA	Sangerseq.
15114321	*C*	*T*	NA	*ADAM23*	intronic	NA	Sangerseq.
15114336	*G*	*C*	NA	*ADAM23*	intronic	NA	Sangerseq.
15115295	*G*	*A*	BICF2P968072	*ADAM23*	intronic	NA	Sequenom
15115366	*G*	*A*	BICF2P968073	*ADAM23*	intronic	NA	Sequenom
15116559	*T*	*C*	NA	*ADAM23*	intronic	NA	Sequenom
15117171	*T*	*C*	BICF2P302559	*ADAM23*	intronic	NA	-
15118890	*A*	*G*	NA	*ADAM23*	intronic	NA	-
15118894	*A*	*G*	NA	*ADAM23*	intronic	NA	Sequenom
15119387	*T*	*C*	NA	*ADAM23*	intronic	NA	Sequenom
15120103	*A*	*G*	NA	*ADAM23*	intronic	NA	-
15120108	*G*	*A*	NA	*ADAM23*	intronic	NA	-
15120524	*T*	*C*	BICF2P1352138	*ADAM23*	intronic	NA	-
15120746	*T*	*C*	NA	*ADAM23*	intronic	NA	-
15120963	-	*TGT*	NA	*ADAM23*	intronic	NA	-
15121756	*T*	*C*	BICF2P86747	*ADAM23*	intronic	NA	Sequenom
15121845	*T*	-	NA	*ADAM23*	intronic	NA	Sequenom
15122108	*G*	*A*	BICF2P86748	*ADAM23*	intronic	NA	-
15123005	*A*	*C*	BICF2P86749	*ADAM23*	intronic	NA	Sequenom
15123950	-	*AA*	NA	*ADAM23*	intronic	NA	Sequenom
15124213	*G*	*A*	BICF2P890779*	*ADAM23*	intronic	NA	Sequenom
15124269	*T*	*C*	BICF2P890780	*ADAM23*	intronic	NA	-
15124474	*T*	*C*	BICF2P890781	*ADAM23*	intronic	NA	Sequenom
15181443	-	*T*	NA	*ADAM23*	intronic	NA	Sequenom

* SNP included in the Canine HD bead array; Bp: base pair position;
Ref.: reference allele; Alt.: alternative allele, NA: not available
